# Complete hybrid genome assembly of clinical multidrug-resistant *Bacteroides fragilis* isolates enables comprehensive identification of antimicrobial-resistance genes and plasmids

**DOI:** 10.1099/mgen.0.000312

**Published:** 2019-11-07

**Authors:** Thomas V. Sydenham, Søren Overballe-Petersen, Henrik Hasman, Hannah Wexler, Michael Kemp, Ulrik S. Justesen

**Affiliations:** ^1^​ Research Unit of Clinical Microbiology, Department of Clinical Research, University of Southern Denmark, Odense, Denmark; ^2^​ Department of Clinical Microbiology, Odense University Hospital, Odense, Denmark; ^3^​ Department of Clinical Microbiology, Lillebaelt Hospital, Vejle, Denmark; ^4^​ Bacteria, Parasites and Fungi, Statens Serum Institut, Copenhagen, Denmark; ^5^​ GLAVA Health Care System and David Geffen School of Medicine, UCLA (University of California, Los Angeles), Los Angeles, CA, USA

**Keywords:** *Bacteroides fragilis*, antimicrobial resistance, genome sequencing, plasmid, Oxford Nanopore, hybrid assembly, insertion sequences

## Abstract

*
Bacteroides fragilis* constitutes a significant part of the normal human gut microbiota and can also act as an opportunistic pathogen. Antimicrobial resistance (AMR) and the prevalence of AMR genes are increasing, and prediction of antimicrobial susceptibility based on sequence information could support targeted antimicrobial therapy in a clinical setting. Complete identification of insertion sequence (IS) elements carrying promoter sequences upstream of resistance genes is necessary for prediction of AMR. However, *de novo* assemblies from short reads alone are often fractured due to repeat regions and the presence of multiple copies of identical IS elements. Identification of plasmids in clinical isolates can aid in the surveillance of the dissemination of AMR, and comprehensive sequence databases support microbiome and metagenomic studies. We tested several short-read, hybrid and long-lead assembly pipelines by assembling the type strain *B. fragilis* CCUG4856^T^ (=ATCC25285=NCTC9343) with Illumina short reads and long reads generated by Oxford Nanopore Technologies (ONT) MinION sequencing. Hybrid assembly with Unicycler, using quality filtered Illumina reads and Filtlong filtered and Canu-corrected ONT reads, produced the assembly of highest quality. This approach was then applied to six clinical multidrug-resistant *B. fragilis* isolates and, with minimal manual finishing of chromosomal assemblies of three isolates, complete, circular assemblies of all isolates were produced. Eleven circular, putative plasmids were identified in the six assemblies, of which only three corresponded to a known cultured *Bacteroides* plasmid. Complete IS elements could be identified upstream of AMR genes; however, there was not complete correlation between the absence of IS elements and antimicrobial susceptibility. As our knowledge on factors that increase expression of resistance genes in the absence of IS elements is limited, further research is needed prior to implementing AMR prediction for *B. fragilis* from whole-genome sequencing.

## Data Summary

Sequence read files [Oxford Nanopore Technologies (ONT) Fast5 files and Illumina fastq files), as well as the final genome assemblies, have been deposited in NCBI/ENA/DDBJ under BioProject accession numbers PRJNA525024, PRJNA244942, PRJNA244943, PRJNA244944, PRJNA253771, PRJNA254401 and PRJNA254455. The fastq format of demultiplexed ONT reads trimmed of adapters and barcode sequences are available at https://doi.org/10.5281/zenodo.2677927. Genome assemblies from the assembly pipeline validation are available at https://doi.org/10.5281/zenodo.2648546. Genome assemblies corresponding to each stage of the process of the assembly are available at https://doi.org/10.5281/zenodo.2661704. Full commands and scripts used are available from GitHub (https://github.com/thsyd/bfassembly), as well as a static version (https://doi.org/10.5281/zenodo.2683511).

**Impact Statement**

Bacterial whole-genome sequencing (WGS) is increasingly used in public health, clinical and research laboratories for typing, identification of virulence factors, phylogenomics, outbreak investigation and identification of antimicrobial-resistance (AMR) genes. In some settings, diagnostic microbiome amplicon sequencing or metagenomic sequencing directly from clinical samples is already implemented and informs treatment decisions. The prospect of prediction of antimicrobial susceptibility based on resistome identification holds promise for shortening the time from sample to report and informing treatment decisions. Databases with comprehensive reference sequences of high quality are a necessity for these purposes. *Bacteroides fragilis* is an important part of the human commensal gut microbiota and is also the most commonly isolated anaerobic bacterium from non-faecal clinical samples, but few complete genome assemblies are available through public databases. The fragmented assemblies from short-read *de novo* assembly often negate the identification of insertion sequences (ISs) upstream of AMR gens, which is necessary for prediction of AMR from WGS. Here, we test multiple assembly pipelines with short-read Illumina data and long-read data from Oxford Nanopore Technologies MinION sequencing to select an optimal pipeline for complete genome assembly of *B. fragilis *. However, *B. fragilis* is a highly plastic genome with multiple inversive repeat regions, and complete genome assembly of six clinical multidrug-resistant isolates still required minor manual finishing for half the isolates. Complete identification of known ISs and resistance genes was possible from the completed genomes. In addition, the current catalogue of *Bacteroides* plasmid sequences is augmented by eight new plasmid sequences that do not have corresponding, complete entries in the National Center for Biotechnology Information database. This work almost doubles the number of publicly available complete, finished chromosomal and plasmid *B. fragilis* sequences paving the way for further studies on AMR prediction, and increased quality of microbiome and metagenomic studies.

## Introduction

*Bacteroides fragilis* is a Gram-negative anaerobic bacterium that is commensal to the human gut, but can act as an opportunistic pathogen; it is the most commonly isolated anaerobic bacteria from non-faecal clinical samples [1]. Antimicrobial-resistance (AMR) rates are increasing for *B. fragilis *, especially for carbapenems and metronidazole, two widely used antimicrobials for treatment of severe infections and anaerobe bacteria [2, 3]. Antimicrobial-susceptibility testing of anaerobes using agar dilution or gradient strip methods can be costly and labour intensive, and despite efforts to validate disc diffusion as a less expensive option, turn-around time will still be least 18 h and validation for individual species will be required [4].

AMR prediction from bacterial whole-genome sequences, from cultured isolates as well as metagenomes, could be implemented in clinical microbiology in the near future, with the potential for improved sample-to-report turnover time and possibly eliminating the need for phenotypical testing for individual species [5–8]. For a few species, prediction of AMR from whole-genome sequencing (WGS) has been validated, but for the majority of clinical relevant species challenges still remain [6, 9, 10].

Based on DNA–DNA hybridization studies, *B. fragilis* can be divided into two DNA homology groups (division I and II), whose ribosomal contents are so different that the two divisions can be distinguished by MS routinely used to identify isolates in clinical laboratories [11]. *B. fragilis* division I isolates carry the chromosomal cephalosporinase gene *cepA,* whilst *B. fragilis* division II isolates harbour the chromosomal metallo-β-lactamase gene *cfiA* (also known as *ccrA*) [12, 13]. The *cfiA* gene can confer resistance to carbapenems, a class of antimicrobials usually reserved for patients with severe sepsis or infections with multidrug-resistant (MDR) bacteria. But expression levels are partly controlled by insertion sequence (IS) elements carrying promotor sequences inserted upstream of the gene and only 30–50 % of clinical isolates that harbour *cfiA* display phenotypically reduced susceptibility to carbapenems [3]. The same pattern of expression control can be observed for genes associated with resistance to metronidazole (*nim* genes) and clindamycin (*erm* genes) [1].

In 2014, we observed that identification of IS elements upstream of known AMR genes in *B. fragilis* was hampered in short-read *de novo* assemblies even though the genes could be identified [14]. This occurred because contigs were often terminated close to the start of the resistance genes, presumably due to the proliferation of multiple copies of the same IS elements throughout the *B. fragilis* genomes. Genome assemblies from short-read sequencing technologies alone most often result in fragmented assemblies, because of repetitive regions and genome elements with multiple occurrences in the chromosomes and plasmids [15, 16]. Therefore, we could not predict AMR phenotypes in *B. fragilis* using only short reads for WGS, since IS element identification is a prerequisite for correct genotype–phenotype associations. Long-read sequencing technologies are increasingly being utilized to increase the contiguity of bacterial genome assemblies, and often result in complete, closed chromosomes and plasmids [17–20]. This provides possibilities for comprehensive identification of IS elements, insights into genome structures, and characterization of other mobilizable elements and associated genes. Complete identification and characterization of plasmids in sequenced isolates would allow for improved analysis of the plasmid-mediated spread of AMR.

Bioinformatic analysis of WGS data depends heavily on high-quality reference databases. Anaerobes make up most of the bacterial human commensal microbiota, but are most likely underrepresented in public databases of whole genomes from cultured isolates. The National Center for Biotechnology Information (NCBI) genome database (accessed 31/03/2019) contains genome sequences of 191 411 bacteria, of which 13 483 are marked as complete assemblies. Only seven of these are *B. fragilis* [21–27]. In comparison, there are 776 assemblies of *Escherichia coli* marked as complete and 398 of *Staphylococcus aureus *. Improving the representation of complete assemblies of *B. fragilis* in the public genome databases will support the development of AMR prediction from WGS, as well as microbiome and metagenomic analysis projects.

The aims of this study were to select an optimal assembly software pipeline for complete, circular assembly of *B. fragilis* and demonstrate the utility of complete assembly for both plasmid identification and comprehensive detection of genes and IS elements associated with AMR. We assembled the *B. fragilis* CCUG4856^T^ (=ATCC25285=NCTC9343) reference strain utilizing long reads generated with the MinION sequencer from Oxford Nanopore Technologies (ONT) and high-quality Illumina short reads, and selected the best assembly pipeline by comparing assemblies to the Sanger-sequenced reference NCTC9343 (RefSeq accession no. GCF_000025985.1). The best assembly pipeline was then applied to six clinical MDR *B. fragilis* isolates from our 2014 study [14].

## Methods

### Culture conditions and DNA extraction

*B. fragilis* CCUG4856^T^ and the six strains described in our previous study were included [14, 21]. Strains were stored at −80°C in beef extract broth with 10 % (v/v) glycerol (SSI Diagnostica), and cultured on solid chocolate agar with added vitamin K and cysteine (SSI Diagnostica) for 48 h in an anaerobic atmosphere at 35 °C. Ten microlitres of culture was transferred to 14 ml saccharose serum broth (SSI Diagnostica) and incubated for 18 h under the same conditions. DNA was then extracted using the Genomic-Tip G/500 kit (Qiagen) following the manufacturers protocol for Gram-negative bacteria and eluted into 5 mM Tris pH 7.5, 0.5 mM EDTA buffer. Quality control was performed by measuring fragment length on a TapeStation 2500 (Genomic DNA ScreenTape; Agilent), purity on a NanoDrop instrument (ThermoFisher Scientific) and concentration on a Qubit instrument (dsDNA BR kit; Invitrogen). The eluted DNA was then stored at −20 °C.

### Illumina library preparation, sequencing and quality control

The strains had previously been sequenced and assembled using Illumina short reads for our previous study [14], but to minimize biological disparities we opted to re-sequence with Illumina using the same DNA extraction prepared for long-read sequencing. Paired-end libraries were generated using the Nextera XT DNA sample preparation kit (Illumina), according to the manufacturer’s protocol. DNA was sequenced on a MiSeq sequencer (Illumina) with 150 bp reads for a theoretical read depth of 100×. Read quality metrics were evaluated using fastqc (https://www.bioinformatics.babraham.ac.uk/projects/fastqc/) and fastp v0.19.6 [28]. Filterbytile from the BBMap package (http://sourceforge.net/projects/bbmap/) was used with default parameters for removing low-quality reads based on positional information on the sequencing flowcell and Trim Galore (http://www.bioinformatics.babraham.ac.uk/projects/trim_galore/), with settings --qual 20 and --length 126, provided additional adapter and quality trimming. fastq files were then randomly down-sampled to <100× crude read depth using an estimated genome size of 5.3 Mb, as higher read depths tend to reduce assembly quality [29].

### Nanopore library preparation and MinION sequencing

Sequencing libraries were prepared using the Rapid Barcoding kit (SQK-RPB004; ONT) following the manufacturers protocol (version RPB_9059_v1_revC_08Mar2018) with SPRI bead clean up (AMPure XT beads; Beckman Coulter) as described. Sequencing was performed as multiplex runs on a MinION connected to a Windows PC with MinKnow v1.15.1 using FLO-MIN106 R9.4 flowcells. Raw Fast5 files were transferred to the Computerome high-performance cluster (https://www.computerome.dk/) for analysis. Four sequencing runs were performed, as the first two runs did not provide enough data for complete assembly of all isolates (see Results).

### Fast5 demultiplexing, base-calling, quality control and filtering

The raw Fast5 files were demultiplexed with Deepbinner v0.2.0 and base-called using Albacore v2.3.3, retaining only those barcodes Deepbinner and Albacore agreed upon for minimal barcode misclassification [30]. Porechop v0.2.4 (https://github.com/rrwick/Porechop) with *--check_reads 100* and *--discard_middle* options was used for adapter and barcode trimming, and read statistics were collected using NanoPlot [31]. Filtlong v0.2.0 (https://github.com/rrwick/Filtlong), with parameters *--min_length 100 --keep_percent 90 --target_bases 500000000,* was used to filter the long reads by either removing the worst 10 % or by retaining 500 Mbs in total, whichever option resulted in fewer reads.

### Assembly validation

To select and validate the optimum assembly pipeline *B. fragilis* CCUG4856^T^ was assembled using a variety of well-known assemblers and polishing tools (Table 1). Each assembler was run with the Filtlong filtered reads as input or the filtered reads corrected with Canu 1.8 (with *--genomSize=5.4* m and *corMinCoverage=* *0* or *coroutCoverage=* *999*). Canu was also tested with the unfiltered reads as input. Hybrid assemblers used the filtered long reads and the filtered, trimmed and down-sampled Illumina reads. Unicycler includes polishing with Racon and Pilon. For assemblers other than Unicycler, Racon polishing with ONT reads was run for one or two rounds, and Pilon was run until no changes were made or for a maximum of six rounds. Racon polishing with Illumina reads was run for one round.

**Table 1.** Genome assemblers and polishing tools tested

Genome assembler and version	Link	Reference
Wtdbg2 v2.3	https://github.com/ruanjue/wtdbg2	[70]
Miniasm v0.3r179	https://github.com/lh3/miniasm	[39, 71]
Flye v2.3.7	https://github.com/fenderglass/Flye	[59, 72]
Canu v1.8	https://github.com/marbl/canu	[73]
SPAdes (including HybridSPAdes) v3.13.0	https://github.com/ablab/spades	[74, 75]
Skesa v2.3.0	https://github.com/ncbi/SKESA	[76]
Unicycler v0.4.7	https://github.com/rrwick/Unicycler	[77]
**Assembly polishing tools**		
Nanopolish v0.10.2	https://github.com/jts/nanopolish	[78]
Racon v1.3.1	https://github.com/isovic/racon	[79]
Pilon v1.22	https://github.com/broadinstitute/pilon	[80]

The original Sanger-sequenced *B. fragilis* NCTC9343 (=CCUG4856^T^) [21] downloaded from NCBI RefSeq (accession no. GCF_000025985.1) was used as a reference sequence for the assembly comparisons and Quast v5.0.2 was used for assembly summary statistics, indel count and K-mer-based completion [32]. busco v3.0.2b (with parameters: *--lineage_path <path to the bacteroidetes_odb9dataset> --mode genome --force*), CheckM v1.0.12 (with default parameters) and Prokka v1.13.3 (with parameters: *--compliant*) were used to assess gene content [33–35]. Average nucleotide identity (ANI) was calculated using software available at https://github.com/chjp/ANI/blob/master/ANI.pl and ale v0.9, which uses a likelihood-based approach to assess the quality of different assemblies based on alignment of Illumina reads, was also used to score the assemblies using default parameters [36, 37]. Ranking of assemblies was based on number of contigs, number of circular contigs, closeness to total length compared to the reference genome, number of local misassembles, number of mismatches per 100 kb, number of indels per 100 kb, ANI, CheckM and busco scores, and the total ale score (a higher score is better). Please see https://github.com/thsyd/bfassembly for full bioinformatics methods.

### Genome assembly of MDR *B. fragilis* isolates

The assembly strategy deemed to produce the highest-quality genome for CCUG4856^T^ was chosen for initial assembly of the six MDR *B. fragilis* isolates. Manual finishing of incomplete assemblies was performed using Bandage for visualization of assembly graphs and blastn searches [38]. Minimap2 and BWA MEM were used to map reads to the assemblies for coverage graphs [39, 40]. Long-read assembly with Flye was compared to the Unicycler assembly, and used to guide and validate the manual finishing results. Circlator’s *fixstart* task was used to fix the start position of the manually finished genomes to be at the *dnaA* gene [41].

The assembled genomes were submitted to NCBI GenBank and annotated with pgap [42]. ABRicate v0.8.10 (https://github.com/tseemann/ABRicate) (with options --minid 40 --mincov 25) was used to screen for AMR genes with the ResFinder (database date 19/08/2018), NCBI Bacterial Antimicrobial Resistance Reference Gene Database (database date 19/09/2018) and card (v2.0.3) databases, supplemented with nucleotide sequences for the multidrug efflux-pump genes *bexA* (GenBank accession no. AB067769.1: 3564…4895) and *bexB* (GenBank accession no. AY375536.1: 4599…5963) [43, 44]. IS elements were identified using ABRicate with data from the IS-finder database (http://www-is.biotoul.fr/; update: 2018/07/25) [45].

### Identification of plasmids and mobile genetic elements

The PLSDB web server (https://ccb-microbe.cs.uni-saarland.de/plsdb/) (data v. 2019_03_05) contains bacterial plasmid sequences retrieved from the NCBI, and was used for screening and identifying putative plasmids sequences [46]. Only hits to accessions from cultured organisms were included. Putative plasmids not identified using PLSDB were evaluated by the read depth relative to the chromosome (higher relative read depth indicates plasmid sequence) and Pfam families covering known plasmid replication domains from table 1 in the 2014 reference by Jørgensen and colleagues [47] were downloaded from the Pfam database (Pfam 32.0; https://pfam.xfam.org/) and used for screening putative plasmids with ABRicate.

## Results

### Sequencing data quality

For Illumina data, a median of 3 465 082 reads [interquartile range (IQR): 3 177 493–5 001 077] were generated for each isolate (Table S1, available with the online version of this article). After filtering, adapter removal and down sampling, a median of 449 022 741 bases (IQR: 433 517 549–530, 57 210) was available per isolate with 87–96 % Q30 bases corresponding to calculated read depths of 75–103 %. The mol% G+C content of the reads for each isolate (median 42.9 mol%, range 42.6–43.3 mol%) were very consistent and within the expected range for the genus *Bacteroides* (40–48 mol%) [48] .

Isolates were sequenced in runs multiplexed with other isolates not included in this study. Based on initial test assemblies using Unicycler without filtering or Canu correction (not shown), it was concluded that data from the first ONT sequencing runs should be supplemented by additional runs to increase the chance of complete assembly of all isolates. Concatenating reads from runs, a median of 75 598 reads (IQR: 50 210–112 065) with a median length of 2938–4393 bases were generated for each isolate (Table S1). Filtering with Filtlong and correction with Canu resulted in a median of 8515 reads (IQR: 6226–10 370) with median lengths of 6181–38 588 bases for each isolate as input for the assemblies.

### Selecting the optimal assembly pipeline

A total of 141 assemblies of *B. fragilis* CCUG4856^T^ was generated using the various assemblers and polishing steps (Table S2). Compared to the reference genome, Unicycler assemblies were of the highest quality (Table 2). Unicycler, with any of the read input options, produced two circular contigs of the expected lengths, and the differences between the various Unicycler assemblies were minimal (Table 3). Assemblies with Canu-corrected reads showed slightly higher genome fractions and ANIs to the reference and fewer mismatches and indels, when compared to Unicycler alone. Unicycler assemblies corrected with Racon using Illumina reads worsened slightly overall with 0.04–0.19 more indels and 0.14–0.25 more mismatches per 100 kbp. Based on this initial evaluation, the assembly pipeline using Canu-corrected reads with default options was chosen (assembly ‘OF.CS’ in Table 3). This would reduce the number of long reads, compared to Canu correction with corMinCoverage=0 or coroutCoverage=999, and thereby lead to a faster run-time for Unicycler.

**Table 2.** Selected quality indicators for the best genome assembly of *B. fragilis* CCUG4856^T^ per assembly pipeline RefSeq accession GCF_000025985.1 was used as a reference. CM, Canu-corrected with option corMinCoverage=0; CO, Canu-corrected with option coroutCoverage=999; CS, Canu-corrected standard settings; OF, ONT reads filtered with Filtlong; PI[*n*], Pilon polishing with Illumina reads, [*n*] rounds; RI, Racon polishing with Illumina reads; RO2, two rounds of Racon polishing with ONT reads. Full results are available in Table S2.

Assembly	No. of contigs	Largest contig	Total length	Mis-assemblies	Genome fraction (%)	Mismatches per 100 kbp	Indels per 100 kbp	ANI	CheckM completeness	busco score: complete and single-copy/ complete and duplicate/ fragment (of 443)	Prokka genes	Prokka rRNA	Prokka tRNA	Total ale score
GCF_000025985.1	2	5 205 140	5 241 700	0	100.000	0	0	100.000	99.26	442/0/1	4439	19	73	−17071758.95
Skesa	46	553 341	5 201 945	3	99.237	0.23	0.15	99.998	99.26	440/2/1	4391	2	62	−20926329.69
SPAdes	23	1 779 941	5 212 217	4	99.396	0.44	0.17	99.987	99.26	440/2/1	4407	3	56	−19676529.39
Canu.OF.CO.RO2.RI.PI3	2	5 247 938	5 350 432	8	99.972	4.94	15.9	99.975	99.26	442/0/1	4634	19	73	−19283611.73
Flye.OF.CS.PI5.RI	5	2 282 650	5 269 269	4	99.917	1.07	6.24	99.978	99.26	441/1/1	4476	19	73	−18222322.23
Miniasm.OF.CM.RO2.PI5	3	5 204 445	5 277 434	2	99.972	5.21	17.75	99.969	98.88	442/0/1	4607	19	73	−17789234.97
Wtdbg2.OF.CO.RO2.PI6.RI	3	5 192 352	5 234 448	7	99.723	3.23	3.04	99.981	99.26	442/0/1	4437	19	73	−18750266.21
SPAdesHybrid.CS	5	3 093 122	5 242 724	7	99.987	1.89	0.53	99.986	99.26	440/2/1	4441	19	73	−18535980.68
Unicycler.OF.CS	2	5 205 133	5 241 693	2	99.972	0.84	0.48	100.000	99.26	442/0/1	4435	19	73	−17200232.52

**Table 3.** Hybrid Unicycler assemblies of *B. fragilis* CCUG4856^T^ RefSeq accession GCF_000025985.1 was used as a reference. CM, Canu-corrected with option corMinCoverage=0; CO, Canu-corrected with option coroutCoverage=999; CS, Canu-corrected standard settings; OF, ONT reads filtered with Filtlong; RI, Racon polishing with Illumina reads. Unicycler performs assembly polishing with Racon (ONT reads) and Pilon. Full results are available in Table S2.

Assembly	Total length (bp)	Largest contig (bp)	Local mis-assemblies	Genome fraction (%)	Mismatches per 100 kbp	Indels per 100 kbp	K-mer-based compl. (%)	K-mer-based misjoins	ANI	Prokka CDSs	Prokka genes	Total ale score
GCF_000025985.1	5 241 700	5 205 140	0	100.000	0	0	100.00	0	100.000	4346	4439	−17071758.95
OF	5 241 602	5 205 042	3	99.970	1.11	0.65	99.96	0	99.999	4343	4436	−17245134.52
OF.RI	5 241 606	5 205 046	3	99.970	1.09	0.67	99.96	3	99.999	4345	4438	−17247815.86
OF.CS	5 241 693	5 205 133	2	99.972	0.84	0.48	99.97	1	100.000	4342	4435	−17200232.52
OF.CS.RI	5 241 698	5 205 138	2	99.972	0.88	0.52	99.96	1	100.000	4346	4439	−17206271.66
OF.CM	5 241 691	5 205 131	2	99.972	0.88	0.5	99.96	1	100.000	4343	4436	−17201292.44
OF.CM.RI	5 241 696	5 205 136	2	99.972	0.95	0.55	99.97	1	100.000	4343	4436	−17193184.79
OF.CO	5 241 693	5,205,133	2	99.972	0.84	0.48	99.97	1	100.000	4342	4435	−17200232.52
OF.CO.RI	5 241 698	5 205 138	2	99.972	0.88	0.52	99.96	1	100.000	4346	4439	−17206271.66

**Fig. 1.**
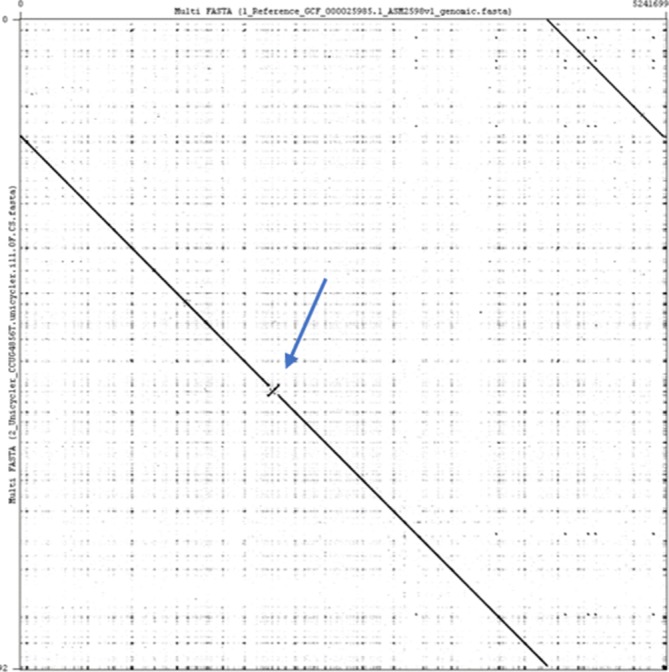
Dot plot matrix of the alignment of the reference assembly and the hybrid Unicycler assembly using Gepard v1.40 [81]. The *B. fragilis* NCTC9343 (RefSeq accession number GCF_000025985.1) reference assembly derived from Sanger sequencing is on the *x*-axis and the hybrid Unicycler assembly on the *y*-axis. On this otherwise near-perfect alignment with high similarity, an 88 045 bp inversion with 100 % ID is observed at nucleotide positions 2 941 962…3 030 006 on the Unicycler assembly (2 005 742…2 093 786 on the reference sequence) (indicated by the blue arrow).

The hybrid Unicycler assembly of CCUG4856^T^ with standard Canu-corrected ONT reads consists of two circular contigs of 5 205 133 and 36 560 bp in length. The plasmid is the same length as plasmid pBF9343 from the reference assembly GCF_000025985.1 and the chromosome is seven bases shorter. Alignments of the Sanger-sequenced assembly GCF_000025985.1 with the hybrid Unicycler assembly show an 88045 bp inversion in the hybrid assembly compared to the Sanger assembly (Fig. 1). This inversion is present in all the best assemblies, including assemblies derived from solely ONT sequences or Illumina sequences (Fig. S1), as well as two additional assemblies of NCTC9343/ATCC25285 from PacBio and Illumina sequences downloaded from NCBI RefSeq (Fig. S2).

### Complete assembly of six MDR isolates

**Fig. 2.**
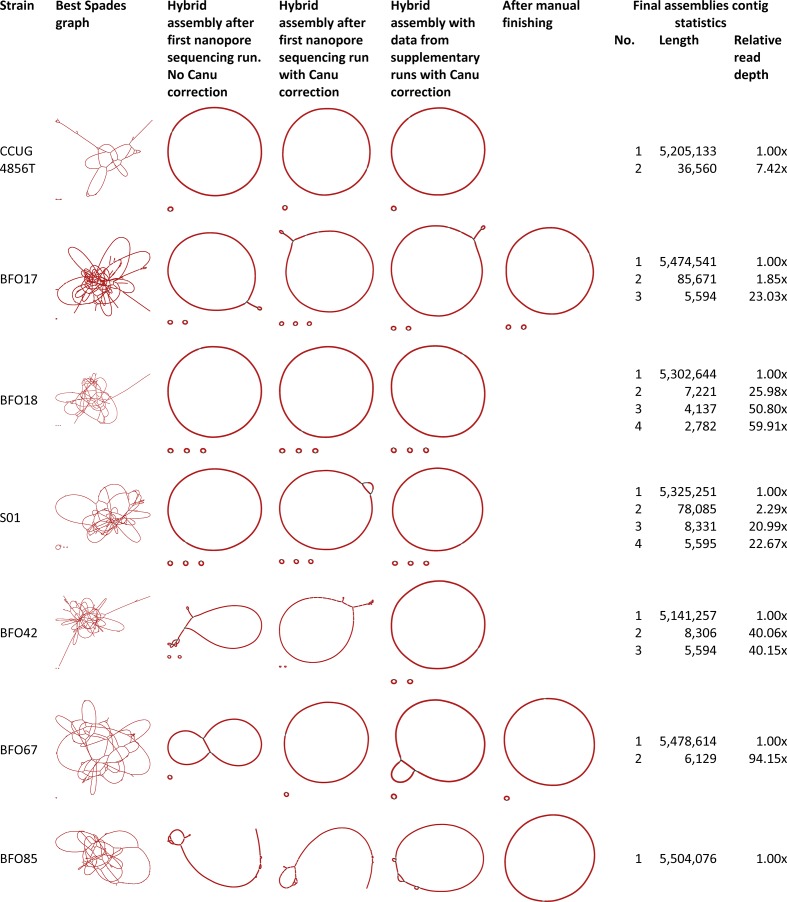
Evolution of genome assemblies with added data and manual finishing. The best SPAdes assembly graphs by Unicycler with short reads only are shown on the far left. Supplying ONT reads improved the assemblies overall, but only three were circularized with singular chromosome contigs with data from the initial MinION sequencing runs. Adding additional ONT data and correcting reads with Canu did not improve assemblies for all isolates. Manual finishing was necessary to finish assemblies for three isolates. Assembly graph images generated with Bandage. Read information can be found in Table S1.

Unicycler, using filtered and trimmed Illumina reads and the Filtlong filtered and Canu-corrected ONT reads from the first sequencing runs, generated complete, continuous, circular assemblies for two of the six isolates (BFO18 and BFO67) (Fig. 2). For the assemblies that were not complete with sequencing data from the first MinION runs, increasing the amount of ONT data resulted in fewer contigs overall, except for BF067, where the additional data from the second sequencing run led to a fragmented assembly and manual finishing was necessary. Performing assembly of isolate S01 without Canu correction of the ONT reads from the first sequencing resulted in a closed chromosome and performing Canu correction of reads resulted in a fragmentation of the chromosome. This was ameliorated by including more ONT data. By manual finishing using read mapping and additional assembly with Flye, the remaining three assemblies were circularized. Chromosomes varied in length from 5 141 257 to 5 504 076 bp. Alignment of ONT and Illumina reads to the chromosome assemblies showed even coverage for both sequencing technologies (Fig. S3). For BFO85, a >100 % relative read depth increase was observed at approximately 25–38 kb. This could represent a 12 kb repeat region that was not resolved in the assembly. Seven (47 %) of the fifteen pgap annotated coding sequences (CDSs) in the 13 kb region were annotated as hypothetical proteins. None of the annotated CDSs represented mobilizable proteins.

### Eleven putative plasmid sequences were identified

A total of 11 putative circular plasmids were identified in the six *B. fragilis* isolates (Table 4). Zero to three putative plasmids were identified per isolate with lengths varying from 2782 to 85 671 bp.

**Table 4.** Putative plasmid sequences of the complete *B. fragilis* assemblies Putative plasmid sequences from the hybrid assemblies of *B. fragilis* CCUG4856^T^ and the six MDR *B. fragilis* isolates were screened using the PLSDB. The best hit to plasmids from cultured isolates is shown. Only three putative plasmids from the MDR *B. fragilis* isolate assemblies could be identified with confident % ID. For most sequences, plasmid replication family proteins were identified in the putative plasmids using ABRicate with a database of sequences downloaded from the Pfam database, strengthening the interpretation that the circularized putative plasmid sequences do in fact represent plasmids harboured by the isolates.

Strain	Sequence	Length (bp)	Relative read depth	mol% G+C	PLSDB results				Plasmid replicon family (% COV, % ID)
					Best hit accession no.	Plasmid hit name	% ID	Length of the sequence of best hit (bp)	
CCUG4856^T^	Chr	5 205 133	1.00×	43.19	–	–	–	–	–
	pBF9343	36 560	7.42×	32.19	NC_006873.1	pBF9343	100	36 560	Rep_3 (100/100)
BFO17	Chr	5 474 541	1.00×	43.51	–	–	–	–	–
	pBFO17_1	85 671	1.85×	36.78	NC_006873.1	pBF9343	80.7	36 560	None
	pBFO17_2	5594	23.03×	39.65	NC_011073.1	pBFP35	99.9	5594	Rep_1 (100/100)
BFO18	Chr	5 302 644	1.00×	43.34	–	–	–	–	–
	pBFO18_1	7221	25.98×	42.32	NC_015168.1	pBACSA02	85.6	19 280	Rep_3 (99.69/99.69)
	pBFO18_2	4137	50.80×	45.40	NC_019534.1	pBFUK1	92.2	12 817	Rep_3 (100.00/98.24)
	pBFO18_3	2782	59.91×	41.45	NC_005026.1	pBI143	94.6	2747	RepL (89.66/49.22)*
S01	Chr	5 325 251	1.00×	43.57	–	–	–	–	–
	pBFS01_1	78 085	2.29×	36.04	NC_006873.1	pBF9343	80.7	36 560	None
	pBFS01_2	8331	20.99×	41.17	NC_015166.1	pBACSA03	95.6	6277	Rep_3 (100.00/97.85)
	pBFS01_3	5595	22.67×	39.62	NC_011073.1	pBFP35	99.9	5594	Rep_1 (100.00/99.48)
BFO42	Chr	5 141 257	1.00×	43.35	–	-	–	–	–
	pBFO32_1	8306	40.06×	43.34	KJ830768.1	pBF69566b	96.0	11 019	RHH_1 (92.94/64.63) Rep_3 (93.64/68.31)
	pBFO32_2	5594	40.15×	39.63	NC_011073.1	pBFP35	99.9	5594	Rep_1 (100.00/99.48)
BFO67	Chr	5 478 614	1.00×	43.85	–	–	–	–	–
	pBFO67_1	6129	94.15×	41.67	NC_011073.1	pBFP35	76.9	5594	Rep_3 (100.00/99.69)
BFO85	Chr	5 504 076	1.00×	43.60	–	–	–	–	–

Chr, Chromosome.

*Annotated as RepA protein in the pgap annotation.

The PLSDB database contains NCBI RefSeq plasmid sequences marked as complete. Three of the eleven putative plasmid sequences were found to match (ID >98 %) a sequence in PLSDB (Table 4). These three all matched the cryptic plasmid pBFP35 [49]. The NCBI nucleotide database was queried using blastn with the remaining unidentified putative plasmid sequences [50]. BFO18 putative plasmid sequence pBFO18_1 (7221 bp) resembles plasmid pIP421, a 7.2 kb plasmid with metronidazole-resistance gene *nimD* and IS*1169*. Partial sequences in NCBI GenBank spanning the *nimD* gene, IS element and *repA* (GenBank accession numbers Y10480.1 and X86702.1) showed 99 % ID (per cent identity) to their alignment to pBFO18_1 (not shown) [51, 52]. Strain S01 putative plasmid sequence pBFS01_2 (8331 bp) showed 99.87 % ID to the 1486 bp partial sequence of *B. fragilis* plasmid pBF388c (GenBank accession number AM042593.1), an 8.3 kb conjugative plasmid harbouring *nimE* and IS*Bf6* [53].

None of the three putative plasmid sequences of strain BFO18 could be identified using the PLSDB, but querying the NCBI nucleotide database using blastn revealed hits for all three. The hits corresponded to circularized sequences [% ID, 99.56–99.96; per cent coverage (% COV), 100] assembled from mobilome metagenomic sequencing of the uncultured caecum content from a rat trapped at Bispebjerg Hospital in Copenhagen, Denmark (a 2 h drive from Odense University Hospital where BFO18 was isolated from a patient’s blood culture) (Table S3) [47, 54]. blastn searches of the remaining unidentified putative plasmids from the other strains did not reveal complete hits.

**Fig. 3.**
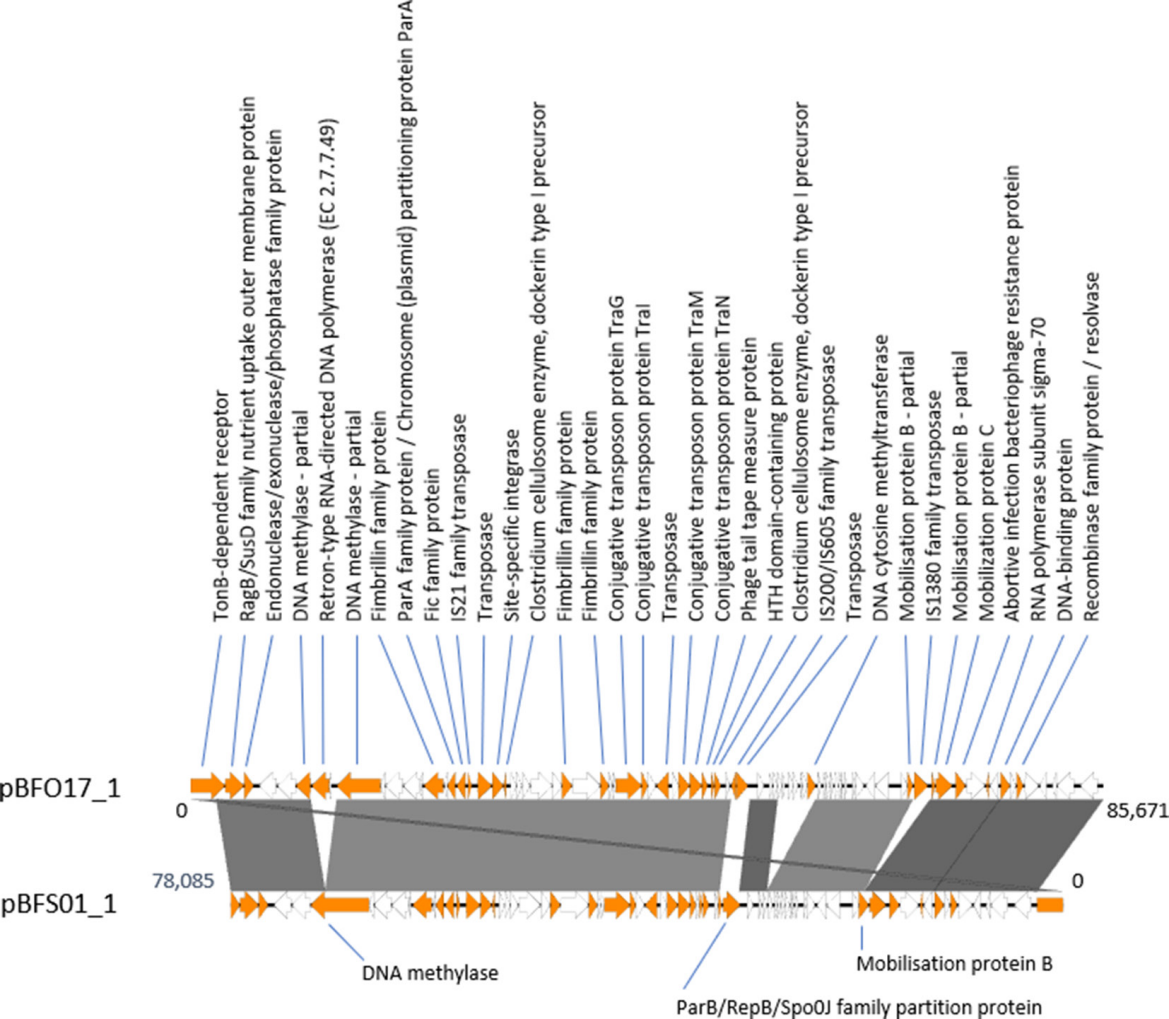
Linear representation of an alignment of putative circular plasmid sequences pBFO17_1 and pBFS01_1 (reverse complement for better visualization) using EasyFig [82]. EasyFig uses blast to identify sequences of similarity. Sequence similarities of >98 % are indicated by full colouring, a darker colour indicates a higher % ID. Products of annotated CDSs are shown. CDSs annotated as hypothetical or domain of unknown function are coloured white. The two sequences show a very high degree of similarity. pBFO17_1 is 7586 bp longer than pBFS01_1. This is mainly due to the insertion of a reverse transcriptase (pBFO17_1, 11367…13034) (disrupting a DNA methylase), the insertion of prophage (from position 56125 to 61162) (identified as an incomplete prophage using phaster [83] and an IS*1380* family-like transposase (67933…69237). The regions pBFO17_1 50711…52501 and pBFS01_1 32248…30304 are not similar. Possibly, the insertion of two transposases in pBFO17_1 have excised most of the ParB-family DNA partitioning protein in the corresponding sequence range in pBFS01_1.

Using ABRicate with the plasmid replication domains collected from the Pfam database, all putative plasmids, except pBF017_1 and pBFS01_1, were found to have recognized replicon domains (Table 4). The circular structures of the two sequences lacking a predicted replication domain were confirmed manually by visually inspecting blastn mapping of ONT sequences longer than 10 kbp to the assembled plasmid sequences with CLC Genomics Workbench 10 (Qiagen). A total of 11 and 22 ONT reads spanned the complete lengths of pBFO17_1 and pBFS01_1, respectively, and contained no other elements. pBFO17_1 and pBFS01_1 demonstrate a degree of similarity of close to 100%, except for an approximate total of 7500 bp transposase and prophage sequences in pBF017_1 (Fig. 3) . No alignment to chromosomal sequences of any of the included *B. fragilis* isolates was observed using progressiveMauve (not shown) [55].

The G+C contents of pBFO17_1 and pBFS01_1 are 36.78 and 36.04 mol%, respectively. These lie within the range for the genus *Bacteroides* but differ from the expected value for *B. fragilis* (43 mol%), which could indicate that the putative plasmids do not originate from *B. fragilis* [56]. After supplementing the pgap annotations with rast annotation [57], 63 % (pBFO17_1) and 59 % (pBFSO1_1) of CDSs remained annotated as hypothetical or as domain of unknown function. Of the annotated CDSs, the majority were associated with mobilizable features, plasmids and phages such as *parA* and *parB*, DNA partitioning proteins, conjugative transposon proteins, transposases, DNA binding motif domain containing proteins, and reverse transcriptase protein. The results above support the assembly data suggesting these two sequences are in fact plasmids.

### Detection of AMR genes and IS elements

We used ABRicate to screen assemblies for AMR genes (ResFinder, NCBI and CARD databases supplemented with sequences for *bexA* and *bexB*) and IS elements (IS-finder database); several AMR genes, possible homologues to known AMR genes and IS elements adjunct to the AMR genes, were detected (Table 5). Of note, isolate BFO17 contains two homologues of the metronidazole-resistance gene *nimJ* (with a 100 % consensus) and two isolates, S01 and BFO85, harbour two homologues of the tetracycline-resistance gene *tetQ*. Homologues to *bexA* and *bexB* were identified with 73.53–99.12 % ID and were all confirmed with blastx searches against the NCBI nr database, as was done in our previous study [14]. Partial hits for *ugd* were observed for several isolates, but with low % ID and % COV, and possibly represent identification of conserved domains, but not *ugd* homologues. Increased expression of the *cfiA* metallo-β-lactamase gene, *nim*-family 5-nitroimidazole genes and *erm* genes is partly regulated through IS elements containing promoter sequences. Full length IS elements could be identified upstream of 11 (79 %) of 14 *cfiA*, *nim* and *erm* genes, and upstream of two of three *cfxA4* genes and the *OXA-347* gene identified in BFO42. The described *B. fragilis* promotors TAnnTTTG (−7) and TG or TTG or TGTG (−33) [58] were searched for manually, but could not be identified upstream of the two *cfiA* genes in isolates BFO67 and BFO85 or the *ermB* gene in BFO85 for which no IS elements could be detected upstream (not shown).

**Table 5.** Antimicrobial susceptibility and resistance genes and IS elements for the six MDR *B. fragilis* strains Identified genes are displayed next to the relevant antimicrobials. Identified IS elements in correct orientation (opposite strand) directly upstream of the genes are included. The % ID and % COV refer to the gene hit. Hits with % ID or % COV <98% were confirmed with blastx searches. The hits for *ugd* represent possible homologues for genes encoding PmrE, which is involved in polymyxin resistance in Gram-negative bacteria. Full ABRicate results with nucleotide positions and information on the IS elements are available in Table S4.

Antimicrobial susceptibility*				AMR genes and IS elements					
Strain	Antimi-crobial	Etest MIC (mg l^−^^1^)	Result	Gene	Upstream IS element	Sequence†	% ID	% COV	Associated resistance to drug class
BFO17	MEM	>32	R	*cfiA11*	IS*614B*	Chr	100.00	99.20	Carbapenem
	IPM	>32	R						
	MTZ	>32	R	*nimJ*	IS*614B*	Chr	99.40	100.00	Nitroimidazole
				*nimJ*	IS*614B*	Chr_c_	99.40	100.00	Nitroimidazole
	CLI	0.094	S						
	TZP	>256	R						
				*tetQ*		Chr	99.34	99.34	Tetracycline
				*cfxA4*		Chr	85.71	100.00	Cephamycin
				*bexB*		Chr_c_	91.21	100.00	Fluoroquinolone
				*bexA*		Chr	73.77	99.02	Fluoroquinolone
BFO18	MEM	>32	R	*cfiA2_1*	IS*Bf12*	Chr	100.00	100.00	Carbapenem
	IPM	16	R				100.00	100.00	
	MTZ	16	R	*nimD*	IS*1169*	S	99.19	100.00	Nitroimidazole
	CLI	6	R	*ermF*‡	IS*4351*	Chr_c_	99.83	72.03	Clindamycin
					IS*Bthe1*‡	Chr_c_	70.97	97.19	
				*erm(F*)§		Chr_c_	99.58	29.71	
				*lnu(AN2*)		Chr_c_	100.00	100.00	Clindamycin
	TZP	>256	R						
				*ugd*		Chr	65.69	53.04	Polymyxin
				*bexA*		Chr_c_	73.60	99.02	Fluoroquinolone
				*bexB*		Chr	91.14	100.00	Fluoroquinolone
				*tet(Q*)		Chr_c_	99.79	100.00	Tetracycline
				*mef(En2*)		Chr_c_	99.83	100.00	Macrolides
S01	MEM	>32	R	*cfiA13_1*	IS*1187*	Chr	99.20	100.00	Carbapenem
	IPM	16	R						
	MTZ	64	R	*nimE*	IS*Bf6*	pBFS01_2	100.00	100.00	Nitroimidazole
	CLI	>32	R	*erm(F*)	IS*1187*	Chr	99.50	100.00	Clindamycin
	TZP	6	S						
				*tetQ*		Chr	90.02	99.95	Tetracycline
				*tet(Q*)		Chr_c_	99.84	100.00	Tetracycline
				*bexB*		Chr_c_	91.06	100.00	Fluoroquinolone
				*bexA*		Chr	74.03	98.80	Fluoroquinolone
BFO42	MEM	0.094	S						
	IPM	0.25	S						
	MTZ	8	R	*nimA*	IS*Bf13*	pBFO32_1	98.64	96.61	Nitroimidazole
	CLI	>256	R	*erm(F*)	IS*613*	Chr	99.50	100.00	Clindamycin
				*lnu(AN2*)		Chr	100.00	100.00	Clindamycin
	TZP	0.38	S						
				*ugd*		Chr	70.38	31.45	Polymyxin
				*cepA-49*		Chr_c_	100.00	100.00	Cephalosporin
				*mef(En2*)		Chr	99.83	100.00	Macrolide
				*ugd*		Chr	71.15	31.11	Polymyxin
				*tetQ*		Chr_c_	100.00	100.00	Tetracycline
				*bexB*		Chr	99.12	100.00	Fluoroquinolone
				*ere(D*)		Chr	96.66	100.00	Erythromycin
				*aadS*		Chr_c_	99.88	100.00	Aminoglycoside
				*OXA-347*	IS*613*	Chr_c_	100.00	100.00	Penicillin, cephalosporin
				*bexA*		Chr	75.09	99.62	Fluoroquinolone
BFO67	MEM	8	R	*cfiA13_1*	None	Chr	100.00	100.00	Carbapenem
	IPM	0.5	S						
	MTZ	0.19	S						
	CLI	0.38	S						
	TZP	2	S						
				*cfxA2*	IS*Bf11*	Chr	99.69	100.00	Cephamycin
				*mef(En2*)		Chr	99.75	100.00	Macrolide
				*lnu(AN2*)		Chr	100.00	100.00	Clindamycin
				*ugd*		Chr_c_	66.76	56.30	Polymyxin
				*tet(Q*)		Chr	100.00	100.00	Tetracycline
				*bexB*		Chr_c_	90.92	100.00	Fluoroquinolone
				*bexA*		Chr	73.90	99.02	Fluoroquinolone
BFO65	MEM	32	R	*cfiA2_1*	None	Chr	100.00	100.00	Carbapenem
	IPM	1	S						
	MTZ	0.25	S						
	CLI	>256	R	*ermB*		Chr_c_	99.19	98.66	Clindamycin
	TZP	2	S						
				*ugd*		Chr	69.84	31.45	Polymyxin
				*tetQ*		Chr_c_	90.02	99.95	Tetracycline
				*aadE*		Chr_c_	100.00	100.00	Aminoglycoside
				*aad9*		Chr_c_	100.00	100.00	Aminoglycoside
				*bexB*		Chr_c_	90.92	100.00	Fluoroquinolone
				*bexA*		Chr	73.53	99.02	Fluoroquinolone
				*cfxA2*	IS*614*	Chr_c_	100.00	100.00	Cephamycin
				*tet(Q*)		Chr_c_	99.84	100.00	Tetracycline

Chr, Chromosome; MEM, meropenem; IPM, imipenem; MTZ, metronidazole; CLI, clindamycin; TZP, piperacillin/tazobactam.

*Results from previously published work following EUCAST (European Committee on Antimicrobial Susceptibility Testing) breakpoints [14].

†﻿A subscript letter C denotes the complement strand.

‡A transposase has inserted itself, splitting the *ermF* gene in two.

### Correlation between identified genes and IS elements and phenotypical resistance

As in our previous study, the *cfiA* gene was identified in the five meropenem-resistant isolates (Table 5). All the *cfiA* genes were found on the chromosomal sequences. Complete IS elements were identified upstream of the *cfiA* genes in BFO17, BFO18 and S01, but not in BFO67 or BFO85. Minimum inhibitory concentrations (MICs) for meropenem and imipenem were lower for these two isolates. *nim* genes (-*A, -D, -E* and -*J*) could be found in the four metronidazole-resistant isolates, all with complete IS elements upstream. Three of the *nim* genes were found on putative plasmids of the respective isolates. The four clindamycin-resistant isolates all carried *erm* genes but for one isolate (BFO85) an upstream IS element was not found. A transposase was inserted in the *ermF gene* in isolate BFO18, splitting it in two, and the same isolate demonstrated a lower clindamycin MIC (6 mg l^−1^) than the other three clindamycin-resistant isolates.

## Discussion

### Hybrid genome assembly produces high-quality *B. fragilis* genomes

The primary aim of this study was to select and validate an assembly method to reliably complete chromosome and plasmid assembly of *B. fragilis* genomes. From 141 assembly variations, a hybrid approach using Filtlong filtered and Canu-corrected ONT reads with quality filtered Illumina reads as input to Unicycler produced a complete, closed assembly of *B. fragilis* CCUG4295^T^ with high similarity to the reference assembly of the original Sanger-sequenced reference assembly. An 88 kb inversion was observed when comparing the two assemblies. Cerdeño-Tárraga and colleagues observed difficulties in resolving certain regions of the Sanger-sequenced assembly of NCTC9343 due to invertible regions with flanking inverted repeat sequences [21]. The observed inversion in the hybrid Unicycler assembly could be due to (a) a superior assembly where the longer ONT reads have overcome the shortcomings of the shorter Sanger sequences, (b) an incorrect assembly by Unicycler, (c) a biological difference that has occurred over time between the strains stored at the National Collection of Type Cultures (NCTC) and the Culture Collection University of Gothenburg (CCUG), or (d) a biological difference that occurred during the culturing of the strain, with dominance of a clone with the inversion, prior to DNA extraction as part of this study. The observations that the inversion is also present in all the best assemblies from this study and assemblies from two other research institutions support the conclusions that the current hybrid Unicycler assembly represents the true orientation of the 88 kb sequence.

### Complete genome assembly of three of the six MDR isolates required manual finishing

The assemblies of BFO18, S01 and BFO42 were completed by Unicycler without manual intervention, but the chromosomes of BFO17, BFO67 and BFO85 could only be closed by performing manual steps. The manual finishing steps are time consuming, difficult to replicate and are easily biased. In order to be implemented in routine clinical laboratories, large scale, automated, complete assembly of prokaryote genomes require robust methods with minimal human interaction. Genome assembly using another long-read assembler, Flye, supported the results of the manual finishing for two of three isolates. Flye is better at resolving repeats than miniasm, the long-read assembler included in the Unicycler pipeline [59]. One option could be to include the long-read assembly from Flye in place of that of miniasm, to guide bridge building for the higher-quality Illumina-only contigs produced in the first steps of Unicycler. To resolve repeats, it is often necessary to have long reads that span the repeat. In prokaryotes, repeats over 10 kb are not unusual and they are often spanned by the ONT reads generated, even by unexperienced users. But repeat regions of up to 120 kb and duplications of 200 kb have been described in some prokaryotes [17, 18, 60]. ONT sequencing runs will routinely result in many reads that span the majority of repeats, but to obtain ONT reads that span specific 120–200 kb repeats in a genome of interest still requires skill and a certain amount of luck. Protocols for ONT sequencing have been described that result in read lengths of over 2 Mb, but this requires skilled and experienced researchers and lab technicians, and demands high amounts of very high quality input DNA and essentially sequencing of only one isolate per MinION flowcell [61].

ONT read depth did not serve as an indicator of whether the Unicycler assemblies would result in closed chromosomal contigs in this study. Final ONT read depth, prior to Filtlong filtering and Canu correction, ranged from 23–371×, but a high read depth alone was not an indicator of closed contigs. The three assemblies BF017, BFO67 and BFO85 required manual finishing to complete the assemblies, and had ONT raw read depths of 99–137×. After Filtlong filtering and Canu correction, the median read lengths were 21 932–29 893 bases and read length N50 was 25 765–34 815 bases for the three isolates (Table S1). Canu correction improved the Unicycler assembly of *B. fragilis* CCUG4856^T^ by nearly all parameters. But whilst Canu correction of the data from the first sequencing run resulted in the complete assembly of BFO67, the assembly of S01 worsened slightly. Increasing the amount of ONT data for BFO67 fragmented the complete chromosome. However, increasing the ONT read depth did decrease the number of contigs per isolate in our study overall.

Defining an optimal approach for complete prokaryote genome assembly is a continuous process, as sequencing technologies and assembly software develop and mature. Ring and colleagues found that Canu correction prior to Unicycler hybrid assembly was superior to other hybrid assembly or long-read assembly approaches for assembly of *Bordetella pertussis* genomes that contain long duplicated regions [18]. Unicycler also performs well in other studies comparing genome assemblers for bacterial genome and plasmid assembly [19]. De Maio and colleagues recently published a preprint comparing hybrid assembly strategies for 20 *Enterobacteriaceae* isolates [20]. In their dataset, simply randomly subsampling ONT reads to an approximate read depth of 100× was slightly superior to applying Canu correction or Filtlong filtering prior to Unicycler assembly. For 85 % of isolates, the expected number of circular contigs were all assembled. For only one additional isolate, Canu correction or Filtlong filtering resulted in the assembly of the expected number of circular contigs. Manual steps, including down sampling ONT reads or removing the Canu correction, are options to consider, if chromosomes are not complete and circularized after initial Unicycler assembly, providing ONT read depth of 100× is available.

We chose to benchmark a selection of widely used genome assemblers for short-read, long-read and hybrid bacterial genome assembly, as well as polishing tools for long-read assemblies, but many other options have been published. Most assemblers and polishing tools were run using default parameters, and it is possible that further optimization of settings for the individual software packages might have improved assemblies further than was demonstrated here. As sequencing technologies and assembly software continues to improve, continued validation of pipelines is advisable. Software such as poreTally provides user-friendly options for benchmarking genome assembly pipelines prior to implementation [62].

### *Bacteroides* plasmids are not well represented in public databases

A secondary aim of this study was to identify plasmids in the hybrid assemblies. Automated tools have been developed and validated for identification of plasmids from genome assemblies or read data, but they are dependant of collated databases of known plasmid sequences. As such, tools such as PlasmidFinder or mlplasmids can be applied for plasmid identification for *Enterobacteriaceae* or *Enterococcus faecium *, but *B. fragilis* is not supported at the time of writing [63, 64]. Therefore, we evaluated putative plasmid sequences by sequence identity and length comparison using the PLSDB webpage, identifying plasmid replication domains, and using circularization and relative coverage as indicators that a sequence represents a plasmid in a given isolate.

Only four of the twelve plasmid sequences from the seven isolates could be identified using the PLSDB and three of these were the same plasmid, pBFP35. Two other putative plasmids, pBFO18_1 and pBFS01_2, were likely plasmids pBF388c and pIP421 based on the partial sequences from these plasmids and plasmid length. This still leaves half of the circularized, putative plasmids unidentified. The two longer putative plasmids, pBFO17_1 and pBFS01_1, displayed a high degree of similarity, a mol% G+C out of the normal range for *B. fragilis* and a relative read depth of double the reads compared to the chromosome. Most annotated CDSs were associated with mobilizable elements, but no known plasmid replication domains could be identified. From the sequencing data alone, we cannot conclude that they represent true plasmids; however, the findings above and manual inspection of long-read mapping support that inference.

There are only 14 complete plasmid sequences from cultured *Bacteroides* isolates in the PLSDB v 2019_03_05, which is based on the NCBI RefSeq database. Many other *Bacteroides* plasmids have been partially described, and some are represented by partial sequences or marked as contig level in the NCBI nucleotide database [65–68]. Metagenomic sequencing and genome assembly projects are expanding the public sequence databases, and screening the NCBI nucleotide database, sequences with a high degree of similarity to the putative plasmid sequences from one patient isolate (BFO18) could be found. These originated from a rat caecum metagenomic plasmid sequencing project from Copenhagen, a few hours’ drive from Odense University Hospital. To understand and perform surveillance of the dissemination of plasmids, there is a need for increased submissions of high quality, annotated and phenotypically validated sequences of bacterial isolates including plasmids. This study adds significantly to the number of complete plasmid sequences associated with *Bacteroides *.

### Complete assembly allows comprehensive identification of resistance determinants in *B. fragilis*

We also intended to comprehensively identify resistance genes and IS elements in the hybrid genome assemblies. Using ABRicate with several resistance gene databases and IS-element nucleotide sequences, the findings of our previous study were confirmed and enhanced. Assemblies from Illumina sequencing alone would only allow partial IS element identification [14]. With the complete assemblies, comprehensive identification of known IS elements upstream of the relevant resistance genes could be completed. In our first study, we used ResFinder with the available database at that time. Additionally, by including several databases, and lowering the % ID threshold, the number of genes identified increased. Lowering the % ID threshold resulted in identification of a possible *cfxA* allele in BFO17, putative *bexA* alleles in several isolates and nucleotide sequences with 66–71 % ID similarity to the *ugd* polymyxin-resistance gene but only 30–56 % COV. The later could possibly be the genes responsible for the inherent polymyxin resistance in *B. fragilis *. Genes with lower than 95 % nucleotide similarity can still encode proteins with 100 % amino acid similarity, as we found for the *bexB* alleles [14]. Therefore, lowering sequence ID thresholds can result in resistance gene alleles that are not (yet) included in nucleotide databases. Conversely, low thresholds can also result in false-positive identifications. Therefore, putative alleles should be analysed manually for proper interpretation. The defaults for running ABRicate (minimum % ID 75 and minimum % COV 0) are probably sufficient for most purposes.

As a result of the complete genome assembly of BFO17, we could now identify two copies of *nimJ*, while only one copy was identified in the short-read draft assembly of the same isolate in the previous study. Husain and colleagues identified the presence of three copies of *nimJ* in strain HMW615, when describing the *nimJ* gene [69]. We confirmed this finding by running ABRicate on the HMW615 assembly as done with the isolates of this study (not shown). Interestingly, rast annotates a third *nim* gene (nucleotide positions 1 359 590…1 360 093) in the Unicycler hybrid assembly of BFO17, and the pgap annotation includes an additional annotation of a pyridoxamine 5'-phosphate oxidase family gene (nucleotide positions 940 032…940 505), the family that includes the *nim* genes. It is possible that one or more novel alleles of the *nim* gene are present in BFO1^7^.

IS elements could be identified upstream of most relevant resistance genes. However, in three cases no IS element was present upstream of a resistance gene, even though the isolates displayed phenotypical resistance associated with increased expression of the specific gene. Known *B. fragilis* promoter sequences could not be identified upstream of the genes ‘missing’ upstream IS elements, but *B. fragilis* promotors are still not completely described, so it is possible there are other unknown variants.

*cfiA*, *nim* and *erm* genes were identified in isolates resistant to meropenem, metronidazole and clindamycin, respectively. However, there was not always co-occurrence of IS elements or identifiable known promotor sequences upstream of the resistance genes and phenotypical resistance. This suggests another unknown mechanism or promoter motifs that ensure sufficient expression of the genes to confer resistance, or the presence of other unknown elements that confer resistance in those isolates.

By selecting an optimal genome assembly strategy for *B. fragilis *, supplemented with minimal manual finishing efforts, and applying this to six MDR isolates, the number of complete *B. fragilis* genomes and plasmids in the public databases has now almost doubled. The future aim of performing AMR prediction based solely on WGS information for *B. fragilis* demands near-complete genomes for identification of IS elements upstream of resistance genes. However, we must caution that the absence of an IS element upstream of *cfiA* does not always correlate with susceptibility to carbapenems. Future studies are needed to address this and utilizing complete genome assemblies for genome-wide association studies is one approach that could be pursued. Technologies that provide a single solution for real-time, high-quality sequencing of long reads will be essential for implementing near real-time diagnostics of infectious diseases and characterization of pathogens.

## Data bibliography

1. Sydenham TV *et al.*, Sequence read files [Oxford Nanopore Technologies (ONT) Fast5 files and Illumina fastq files], as well as the final genome assemblies, have been deposited in NCBI/ENA/DDBJ under BioProject accession numbers PRJNA525024, PRJNA244942, PRJNA244943, PRJNA244944, PRJNA253771, PRJNA254401 and PRJNA254455 (2019).

2. Sydenham TV *et al.,* fastq format of demultiplexed ONT reads trimmed of adapters and barcode sequences, https://doi.org/10.5281/zenodo.2677927 (2019).

3. Sydenam TV *et al.,* Genome assemblies from the assembly pipeline validation, https://doi.org/10.5281/zenodo.2648546 (2019).

4.Cerdeño-Tárraga AM *et al*., Reference assembly of *B. fragilis* CCUG4856^T^, https://www.ncbi.nlm.nih.gov/assembly/GCF_000025985.1/ (2005).

5. Sydenham TV *et al*., Genome assemblies corresponding to each stage of the process of the assembly of the six MDR isolates, https://doi.org/10.5281/zenodo.2661704.

6. El-Gebali S *et al*., Sequences of the plasmid replication domain families were downloaded from the Pfam database (https://pfam.xfam.org/). Specifically, the full-length sequences for all sequences in the full alignments were downloaded for: PF01051, PF01402, PF01446, PF01719, PF01815, PF02486, PF03090, PF03428, PF04796, PF05732, PF06504, PF06970, PF07042 and PF10134 (2019).

## Supplementary Data

Supplementary File 1

Supplementary File 2
